# MiR‐130a‐3p regulates neural stem cell differentiation in vitro by targeting *Acsl4*

**DOI:** 10.1111/jcmm.17285

**Published:** 2022-04-16

**Authors:** Wen Li, Bo‐Quan Shan, He‐Yan Zhao, Hui He, Mei‐Ling Tian, Xiang Cheng, Jian‐Bing Qin, Guo‐Hua Jin

**Affiliations:** ^1^ 66479 Department of Human Anatomy Institute of Neurobiology Nantong University Nantong China; ^2^ 66479 Co‐Innovation Center of Neuroregeneration Nantong University Nantong China; ^3^ Key Laboratory of Neuroregeneration of Jiangsu and Ministry of Education Nantong China

**Keywords:** *Acsl4*, Akt/PI3K signalling pathway, differentiation, MiR‐130a‐3p, neural stem cells

## Abstract

In the adult mammalian brain, neural stem cells (NSCs) are the precursor cells of neurons that contribute to nervous system development, regeneration, and repair. MicroRNAs (miRNAs) are small non‐coding RNAs that regulate cell fate determination and differentiation by negatively regulating gene expression. Here, we identified a post‐transcriptional mechanism, centred around miR‐130a‐3p that regulated NSC differentiation. Importantly, overexpressing miR‐130a‐3p promoted NSC differentiation into neurons, whereas inhibiting miR‐130a‐3p function reduced the number of neurons. Then, the quantitative PCR, Western blot and dual‐luciferase reporter assays showed that miR‐130a‐3p negatively regulated acyl‐CoA synthetase long‐chain family member 4 (*Acsl4*) expression. Additionally, inhibition of *Acsl4* promoted NSC differentiation into neurons, whereas silencing miR‐130a‐3p partially suppressed the neuronal differentiation induced by inhibiting *Acsl4*. Furthermore, overexpressing miR‐130a‐3p or inhibiting *Acsl4* increased the levels of p‐AKT, p‐GSK‐3β and PI3K. In conclusion, our results suggested that miR‐130a‐3p targeted *Acsl4* to promote neuronal differentiation of NSCs via regulating the Akt/PI3K pathway. These findings may help to develop strategies for stem cell‐mediated treatment for central nervous system diseases.

## 1 INTRODUCTION

Neurodegenerative diseases (NDs) are age‐related conditions characterized by the specific subsets of neurons functional deterioration and neuronal cell death, resulting in nervous system dysfunction.^1^, ^2^ Although there have been many studies on the causes and mechanisms of NDs, the treatments currently available remain limited. Neural stem cells (NSCs) are characterized by their ability to develop into different neural lineages, while maintaining self‐renewal capacity, and are crucial for development, regeneration and repair of the nervous system.^3^, ^4^

MicroRNAs (miRNAs) are a class of well‐conserved, small non‐coding RNA (~22 nucleotides) molecules that have been shown to be negative regulators of gene expression at the post‐transcriptional level.^5^, ^6^ Currently, 70% of the known microRNAs have been reported to be expressed in the brain, where they participate in proliferation, neuronal development, synaptic plasticity and neuronal differentiation.^7^, ^8^, ^9^ For example, Chen et al.^10^ demonstrated that miR‐331‐3p and miR‐9‐5p, respectively, targeted Sqstm1 and Optn, regulating autophagic activity and amyloid plaque formation. Wang et al.^11^ revealed that miR‐26a was upregulated in glioma, and enhanced proliferation and angiogenesis of human brain microvascular endothelial cells by targeting PTEN.

Research showed that miR‐130a‐3p regulates the progression of many different malignancies. Tian et al.^12^ found that miR‐130a‐3p was significantly downregulated in oesophageal cancer cell lines, and was further suppressed during oesophageal cancer metastasis via SMAD4. Wang et al.^13^ observed that reduced malignant behaviours and temozolomide resistance in glioblastoma multiforme through downregulation of its target gene Sp1. Zhong et al.^14^ indicated that miR‐130a‐3p expression was suppressed in breast cancer, which was correlated with inhibited proliferation, migration and invasion by targeting SATB1. Additionally, miR‐130a‐3p is also weakly expressed in diabetes, and upregulating miR‐130a‐3p ameliorated the senescence of renal tubular epithelial cells induced by high glucose levels.^15^ Yang et al.^16^ found that exosomal miR‐130a‐3p binding to SIRT7 mRNA promoted osteogenic differentiation of adipose‐derived stem cells in a process that involved the Wnt/β‐catenin pathway. A previous report of miR‐130a‐3p expression in the dorsal root ganglia suggests a mechanism that could indirectly control VEGFR‐2 expression in the peripheral nervous system.^17^

In this study, we identified that miR‐130a‐3p overexpression induced neuronal differentiation of NSCs, while miR‐130a‐3p knockdown inhibited the differentiation of NSCs. Furthermore, luciferase reporter assays demonstrated that acyl‐CoA synthetase long‐chain family member 4 (*Acsl4*) was a miR‐130a‐3p target gene. Downregulation of *Acsl4* promoted the differentiation of NSCs, and neuronal differentiation was decreased by miR‐130a‐3p expression potentially through altered AKT/PI3K signalling pathway. These findings indicated that miR‐130a‐3p may play an important role in regulating NSC differentiation by targeting *Acsl4*. The results of our findings may provide novel insight for the development of stem cell‐mediated treatment for NDs.

## 2 MATERIALS AND METHODS

### 2.1 Animals

Pregnant Sprague‐Dawley (SD) rats and adult SD rats (220–250 g) were purchased from the Laboratory Animal Center of Nantong University (license No. SYXK (Su) 2017–0046). All experimental protocols were approved by the Animal Ethics Committee of Nantong University. All efforts were made to minimize the number and suffering of animals used in this study.

### 2.2 Cell culture

NSCs were prepared from embryonic day (E)15 Sprague‐Dawley rat embryos as previously described.^18^ In total, 20 pregnant rats were used for this study. To isolate and culture NSCs, embryos were removed by caesarean section, and the hippocampus was rapidly mechanically dissociated into a single‐cell suspension. The single‐cell suspension was maintained in flasks containing Dulbecco's modified Eagle's medium/F12 (1:1; Gibco, Grand Island, NY, USA) containing 2% B27 (Gibco), epidermal growth factor (20 ng/mL; Sigma‐Aldrich, St. Louis, MO, USA), and basic fibroblast growth factor (20 ng/mL; Sigma‐Aldrich). Cells were incubated at 37°C and 5% CO_2_ in a humidified incubator. After 5 d, primary neurospheres were dissociated into single cells using trypsin (Gibco). For Nestin/Ki67 analysis, cell suspensions were plated at a density of 1×10^5^ cells/mL on poly‐lysine‐coated coverslips in 24‐well plates (adherent condition) containing NSCs medium. For NSC differentiation, cells were cultured in differentiation medium, which contained 2% foetal bovine serum (FBS, Gibco). Then, cells were plated at a density of 1 × 10^5^ cells/mL in 24‐well plates (adherent condition) or 5 × 10^5^ cells/mL in 6‐well plates.

### 2.3 Transfection

NSCs at 60%–80% confluence were transiently transfected using Lipofectamine3000 (Invitrogen, Carlsbad, CA, USA) according to the manufacturer's instructions. miRNA compounds miR‐130a‐3p/ negative control (NC) mimics and miR‐130a‐3p/NC inhibitor) were purchased from RiboBio Co., Ltd. (Guangzhou, China). The small interfering (si)‐Acsl4, negative control siRNA, pcDNA3.1‐Acsl4 plasmid, and negative control pcDNA3.1 vector plasmid were purchased from (Genechem, Shanghai, China). The Acsl4 plasmid was subcloned into the pcDNA3.1 vector to overexpress ACSL4. The following siRNA sequences were used: (sense) 5’‐GCTCTGTCACGCACTTCGA‐3’ and (antisense) 3’‐UCGAAGUGCGUGACAGAGC‐5’.

### 2.4 RNA isolation and quantitative real‐time PCR

Total RNA from cells or tissues was extracted using TRIzol reagent (Vazyme Biotech, Nanjing, China). cDNA was synthesized with 1 μg total RNA using the HiScriptII Reverse Kit (Vazyme Biotech) according to the manufacturer's instructions. The AceQ real‐time quantitative polymerase chain reaction Kit (Vazyme Biotech) was used for real‐time quantitative RT‐qPCR assays. Relative mRNA expression levels were normalized to the level of Gapdh. The primer sets used in this study are listed in Table S1.

### 2.5 Flow cytometry

The proliferation of cells was measured by a Cell‐Light 5‐ethynyl‐2ʹ‐deoxyuridine (EdU) Apollo567 In Vitro Kit (Ribobio) was used following the manufacturer's protocols, as previously reported (Zheng et al., 2019).^19^ Briefly, the cells were exposed to 50 μM EdU medium for 2 hours, and then were washed and fixed with 4% paraformaldehyde for 30 minutes. Following washing with PBS, the cells were permeabilized and blocked with PBS containing 0.5% Triton X‐100 for 10 minutes. After washing the cells with PBS, the cells were centrifuged and resuspended in 500 µL of PBS.

For cell cycle analysis, cells were harvested and fixed in ice‐cold 75% ethanol at 4°C overnight. Following washing with PBS, the cells were stained with propidium iodide/RNase Staining Buffer (0.5mL/1 × 10^6^ cells) and incubate for 15min at room temperature (BD Biosciences, San Diego, CA, USA).

For cell differentiation assays, cells were trypsinized and collected, washed twice with ice‐cold phosphate‐buffered saline (PBS), and fixed in 1× Fix/Perm Buffer at 4°C for 50 min. After washed with 1× Perm/Wash Buffer twice, the samples were incubated with APC‐conjugated anti‐Tuj1 antibody, APC‐conjugated anti‐GFAP antibody or APC‐conjugated IgG2A Control (5μL/test) (BD Biosciences) (Figure S2) at 4°C for 2 h. After washing the cells with 1× Perm/Wash Buffer twice, the cells were centrifuged and resuspended in 350 µL of flow cytometry stain buffer. Stained cells were assessed using a FACSCalibur Instrument (BD Biosciences).

### 2.6 Immunofluorescence

Cells were fixed with 4% paraformaldehyde for 15 minutes, washed twice with PBS, and then blocked with 0.3% Triton X‐100 containing 10% goat serum for 2 h. After incubation with primary antibodies at 4°C overnight, samples were incubated with the appropriate Alexa Fluor 488 or 594 conjugated secondary antibodies (Invitrogen) for 2 h at room temperature. Nuclei were counterstained with Hoechst 33342 (Sigma‐Aldrich; 1:1,000). Primary antibodies included mouse anti‐Nestin (Millipore, Billerica, MA, USA; 1:200), rabbit anti‐Ki67 (Abcam, Cambridge, MA, USA; 1:200), mouse anti‐MAP2 (Millipore; 1:1000), rabbit anti‐GFAP (Millipore; 1:1000), rabbit anti‐ChAT (Millipore; 1:1000) and mouse anti‐Cnp (Millipore; 1:200). Images of stained cells were captured using an EVOS FL Auto (Invitrogen) microscope, and results are expressed as percentages.

### 2.7 Western blot

Total protein was extracted with cell lysis buffer (Solarbio, Beijing, China). Proteins were separated by 10% SDS‐PAGE and then transferred to nitrocellulose membranes (Millipore). The membranes were blocked with 5% non‐fat milk (Sangon Biotech, Shanghai, China) for 2 h, then incubated with primary antibodies overnight at 4°C, and finally incubated with HRP‐conjugated secondary antibodies for 2 h at room temperature. Next, immunoreactive bands were detected using an enhanced chemiluminescence kit (Bio‐Rad, Hercules, CA, USA). β‐Actin was used as a loading control. The primary antibodies included mouse anti‐MAP2 (Millipore; 1:1000), mouse anti‐Tuj1 (Millipore; 1:1000), rabbit anti‐GFAP (Millipore, 1:1000), rabbit anti‐*Acsl4* (Abcam; 1:1000), rabbit anti‐Akt (Cell Signaling Technology, Danvers, MA, USA; 1:1000), rabbit anti‐phospho‐Akt (Cell Signaling Technology; 1:1,000), rabbit anti‐PI3K (Abcam; 1:1000) and mouse anti‐β‐actin (Abcam; 1:1000).

### 2.8 Dual‐luciferase reporter assay

The pMIR‐target 3′UTR‐luciferase, pMIR‐target 3′UTR mut‐luciferase, and Renilla pRL‐SV40 control vector were synthesized by Genechem Company (Shanghai, China). Then, 293T cells were transfected with the pMIR‐target 3′UTR‐luciferase, pMIR‐target 3ʹ‐UTR mut‐luciferase, Renilla pRL‐SV40 control vector, miR‐130a‐3p mimics or miRNA negative control using Lipofectamine 3000 (Invitrogen). The relative luciferase activities were analysed using the dual‐luciferase assay (Promega, Madison, WI, USA) 72 h after transfection, which was computed by the ratio of firefly luciferase activity to Renilla.

### 2.9 Statistical analysis

All results are displayed as the mean ± SEM using data from at least three separate experiments. GraphPad Prism 6 (GraphPad Software, San Diego, CA, USA) was used to analyse the results. Differences between corresponding groups were evaluated using a two‐tailed Student's t‐test or one‐way analysis of variance followed by Tukey's post hoc test. *p* < 0.05 was considered to represent statistically significant differences.

## 3 RESULTS

### 3.1 Effect of miR‐130a‐3p on NSCs proliferation

**FIGURE 1:**
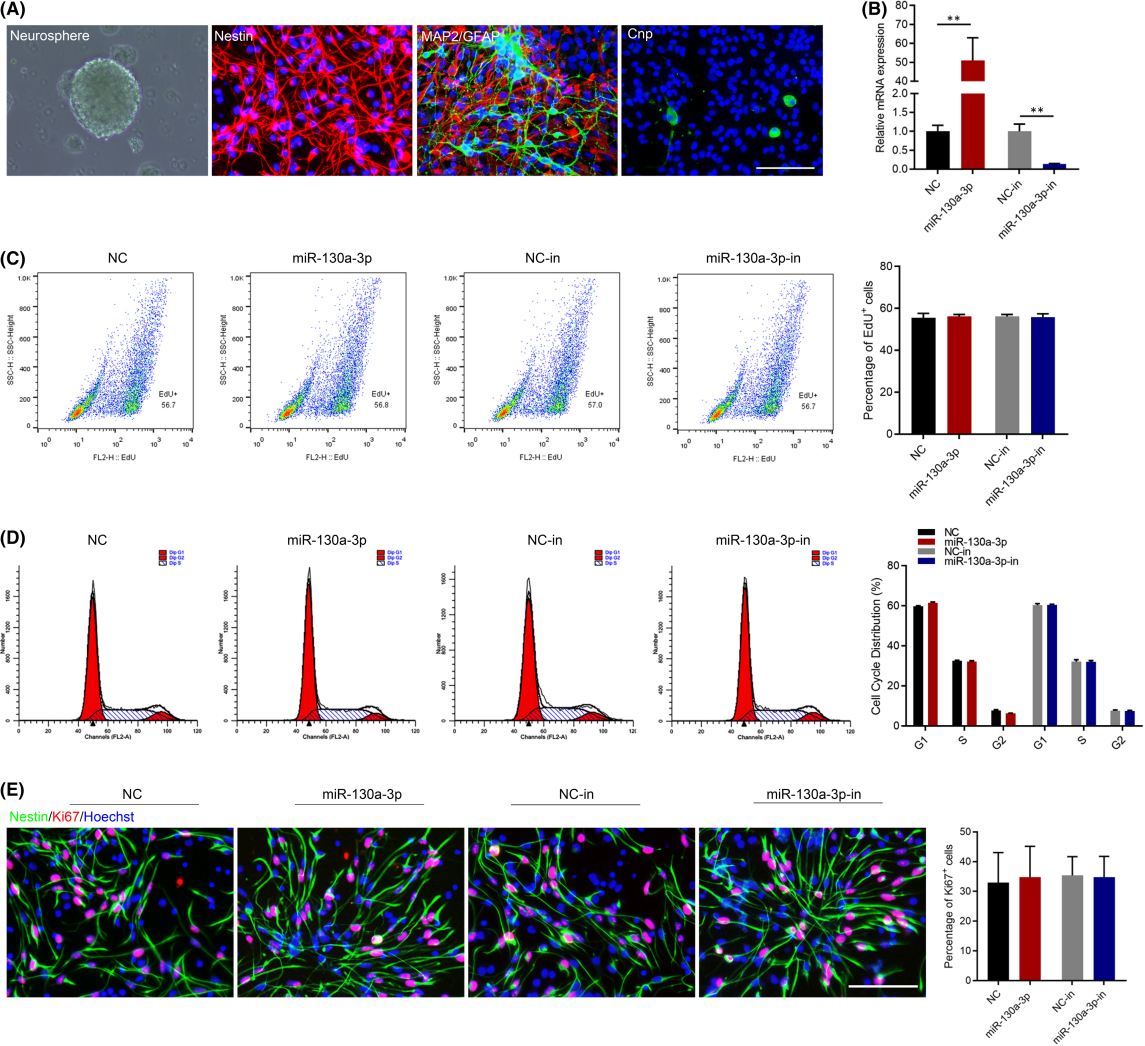
Effects of miR‐130a‐3p on NSC proliferation. (A) To identify NSCs, the expression of Nestin, MAP2, GFAP, and Cnp were detected by Immunofluorescence staining. Scale bars: 100 μm. (B) To study the effect of miR‐130a‐3p on NSCs, they were transfected with miR‐130a‐3p/NC mimics and miR‐130a‐3p/NC inhibitor for 24 h. RT‐qPCR analysis was performed, and normalization to the levels of *U6*. The results are described as the mean ±SEM at least three separate experiments. **p < 0.01, vs. indicated group. (two‐tailed paired Student's *t*‐test). The proliferation of NSCs transfected with miR‐130a‐3p/NC mimics and miR‐130a‐3p/NC inhibitor for 24 h, as detected by EdU assay (C), flow cytometry assay (D) and Immunofluorescence staining (E). Scale bars: 100 μm. The results are described as the mean ± SEM at least three separate experiments

The P1 neurospheres were pictured, and the purity of NSCs was verified by immunofluorescence staining of Nestin, a NSCs marker. To confirm the multilineage differentiation potency, cells were incubated with antibodies against MAP2, GFAP and Cnp on the 7th day after NSC differentiation (Figure 1A), which were markers of neuron, astrocyte and oligodendrocyte, respectively. To determine the effects of miR‐130a‐3p on the proliferation of NSCs, we transfected vector controls and miRNA into NSCs. We found that the expression level of miR‐130a‐3p was significantly upregulated after overexpression of miR‐130a‐3p. However, after inhibition of miR‐130a‐3p, the expression of miR‐130a‐3p was significantly downregulated (Figure 1B). EdU incorporation assays demonstrated that neither overexpression of miR‐130a‐3p or inhibition of miR‐130a‐3p altered the number of EdU positive cells (Figure 1C). Consistent with these results, miR‐130a‐3p had no effect on cell cycle progression (Figure 1D). Immunofluorescence staining of Ki67 also showed that miR‐130a‐3p had no effect on the proliferation of NSCs (Figure 1E).

### 3.2 miR‐130a‐3p promoted neuronal differentiation of NSCs

**FIGURE 2:**
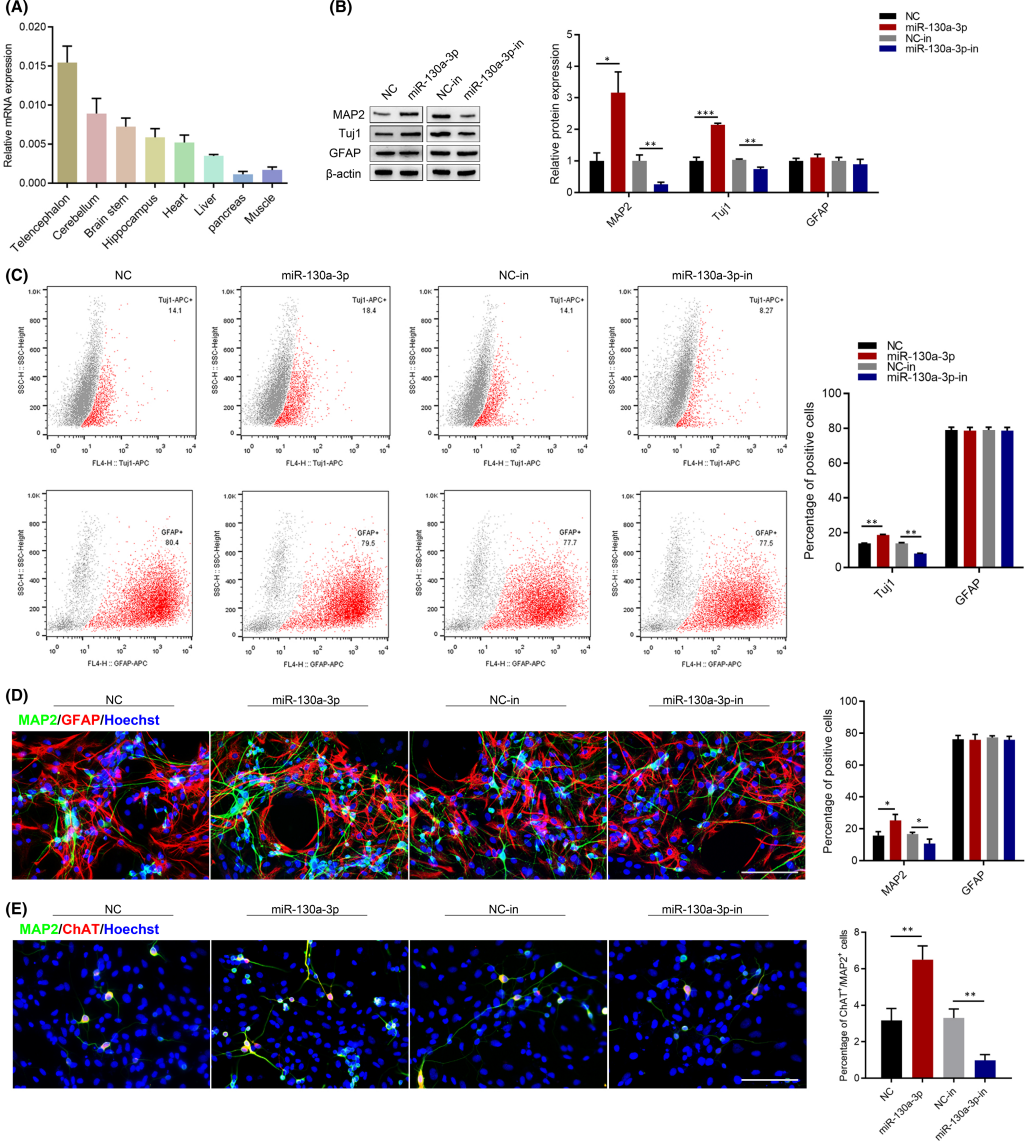
Effects of miR‐130a‐3p on NSC differentiation. (A) miR‐130a‐3p expression in different tissues of adult SD rat was analysed performing qRT‐PCR using Gapdh as endogenous control. The results are described as the mean ± SEM at least three separate experiments. NSCs were transfected with miR‐130a‐3p/NC mimics and miR‐130a‐3p/NC inhibitor for 7d. (B) Western blot was performed to detect the expression of the differentiation markers, MAP2, Tuj1 and GFAP. (C) The percentage of Tuj1 and GFAP positive cells were detected by flow cytometry assay. (D) Immunofluorescence analysis of MAP2(green) and GFAP (red) positive cells. Scale bars: 100 μm. (E) Immunofluorescence analysis of MAP2(green) and ChAT (red) positive cells. Scale bars: 100 μm. The results are described as the mean ± SEM at least three separate experiments. *p < 0.05, **p < 0.01 vs. indicated group. (two‐tailed paired Student's *t*‐test)

We extracted RNA from Telencephalon, cerebellum, brain stem and hippocampus, heart, liver, pancreas and muscle of adult SD rats, and then carried on RT‐qPCR analysis. As shown in Figure 2A, miR‐130a‐3p was expressed significantly higher in nervous tissues compared with other tissues. To determine the effects of miR‐130a‐3p on NSC differentiation into neurons, the dose‐dependent was detected 24 h after cell transfection. The time‐dependent effect of miR‐130a‐3p was detected 24 h, 48 h and 72 h after cell transfection by RT‐qPCR assays. The RT‐qPCR results showed that miR‐130a‐3p significantly increased *Map2*, *Tuj1*, *Neurod1* and *Neun* expression (Figure S1). Similarly, miR‐130a‐3p overexpression remarkably increased MAP2 and Tuj1 levels compared with the NC group, while that inhibition of miR‐130a‐3p suppressed MAP2 and Tuj1 expression 7 d after cell transfection. Moreover, there were no changes in GFAP expression (Figure 2B). Flow cytometry and immunofluorescence showed that the percentage of neurons was notably increased in the miR‐130a‐3p group compared with NC group. In contrast, the percentage of neurons was decreased when miR‐130a‐3p was inhibited. In addition, there were no changes in the number of astrocytes (Figure 2C,D). To explore whether miR‐130a‐3p could induce NSCs to differentiate into specific neurons, the expression of ChAT, an acetylcholine‐synthesizing enzyme was detected 7 d after cell transfection. ChAT is responsible for synthesizing ACh, which plays a crucial role learning and memory function.^20^ Immunofluorescence staining of MAP2 and ChAT differentiated cells indicated that there were more MAP2 and ChAT double positive cells in miR‐130a‐3p group, while less in miR‐130a‐3p‐in group (Figure 2E). Our results revealed that miR‐130a‐3p facilitated differentiation of NSCs into neurons.

### 3.3 miR‐130a‐3p regulated *Acsl4* expression in NSCs

**FIGURE 3:**
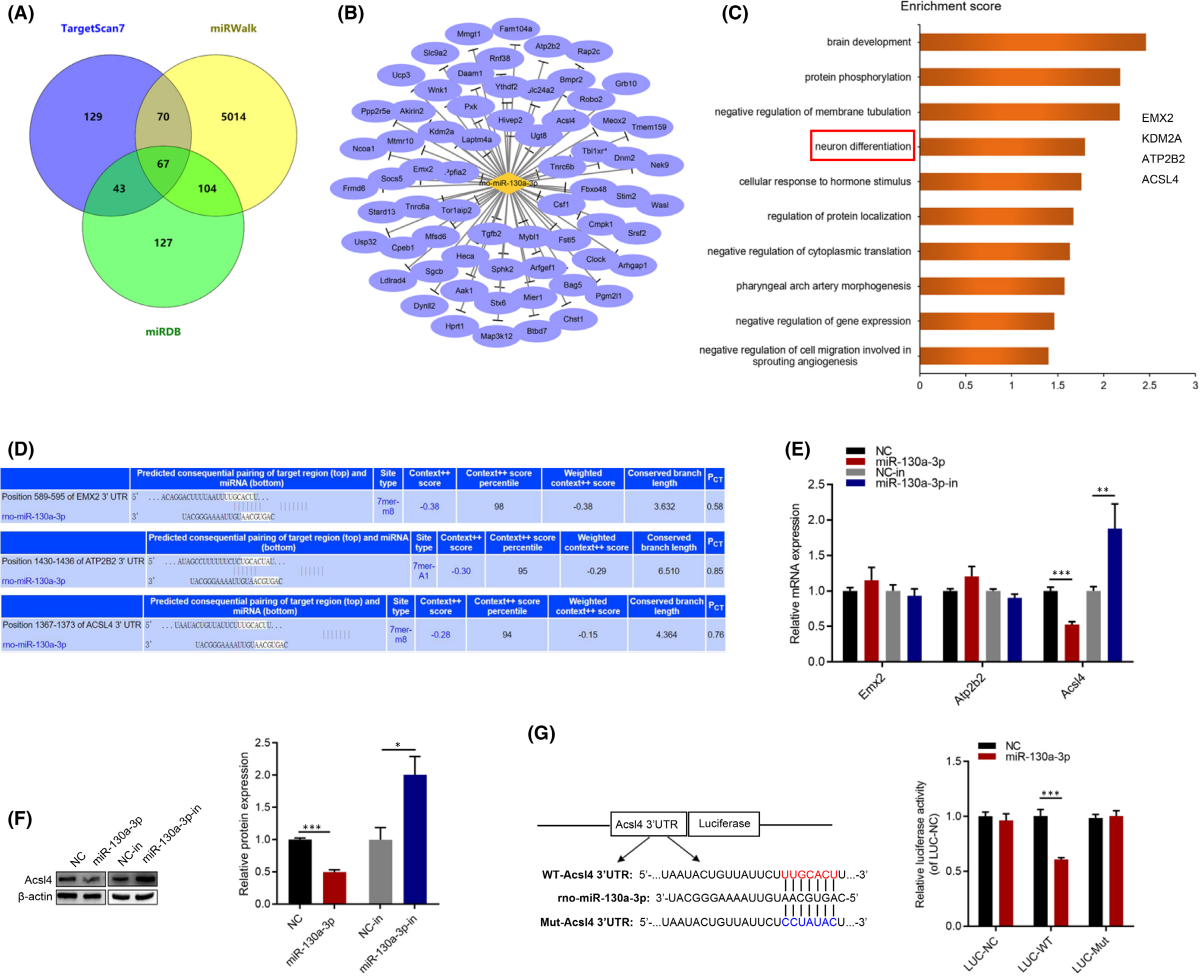
miR‐130a‐3p targets *Acsl4* in NSCs. (A) Predicted target genes of the miR‐130a‐3p detected by bioinformatic analysis. (B) Network of miR‐130a‐3p and their 67 predicted target genes. Diamond nodes represented miR‐130a‐3p and ellipse nodes represented target genes. (C) Gene Ontology analysis of predicted target genes. (D) Predict the results of target genes through the TargetScan7.2 website. (E) NSCs were transfected with miR‐130a‐3p/NC mimics and miR‐130a‐3p/NC inhibitor for 24 h. The mRNA expression of *Emx2*, *Atp2b2* and *Acsl4* was measured by RT‐qPCR, and normalization to the levels of *Gapdh*. (F) The protein expression of *Acsl4* was measured by western blot analyses. (G) Diagram of the potential associating site in the 3ʹ‐UTR region of *Acsl4* with miR‐130a‐3p. Cells were harvested after 72 h and the dual‐luciferase reporter assay system was used to measure luminous intensity. The results are described as the mean ± SEM at least three separate experiments. *p < 0.05, **p < 0.01, ***p < 0.001 vs. indicated group. (two‐tailed paired Student's *t*‐test)

To explore the molecular mechanisms of miR‐130a‐3p, bioinformatic databases including TargetScan7.2, miRDB and miRWalk2.0 were used to screen possible binding sites of miR‐130a‐3p (Figure 3A,B). The 67 predicted target genes were further analysed in the DAVID 6.7, and Gene Ontology (GO) databases, which showed that *Emx2*, *Kdm2a*, *Atp2b2* and *Acsl4* were enriched during neuron differentiation (Figure 3C). The TargetScan7.2 website showed that the context score percentile of *Emx2*, *Atp2b2* and *Acsl4* were >90 (Figure 3D). Using RT‐qPCR and Western blot, we found that *Acsl4* mRNA levels were significantly downregulated after miR‐130a‐3p overexpression, whereas that inhibition of miR‐130a‐3p increased *Acsl4* expression in NSCs (Figure 3E,F). Next, dual‐reporter assays were performed to determine whether *Acsl4* was a direct target of miR‐130a‐3p. These results showed that miR‐130a‐3p mimic significantly inhibited luciferase expression (Figure 3G).

### 3.4 miR‐130a‐3p regulated NSC differentiation by targeting *Acsl4*

**FIGURE 4:**
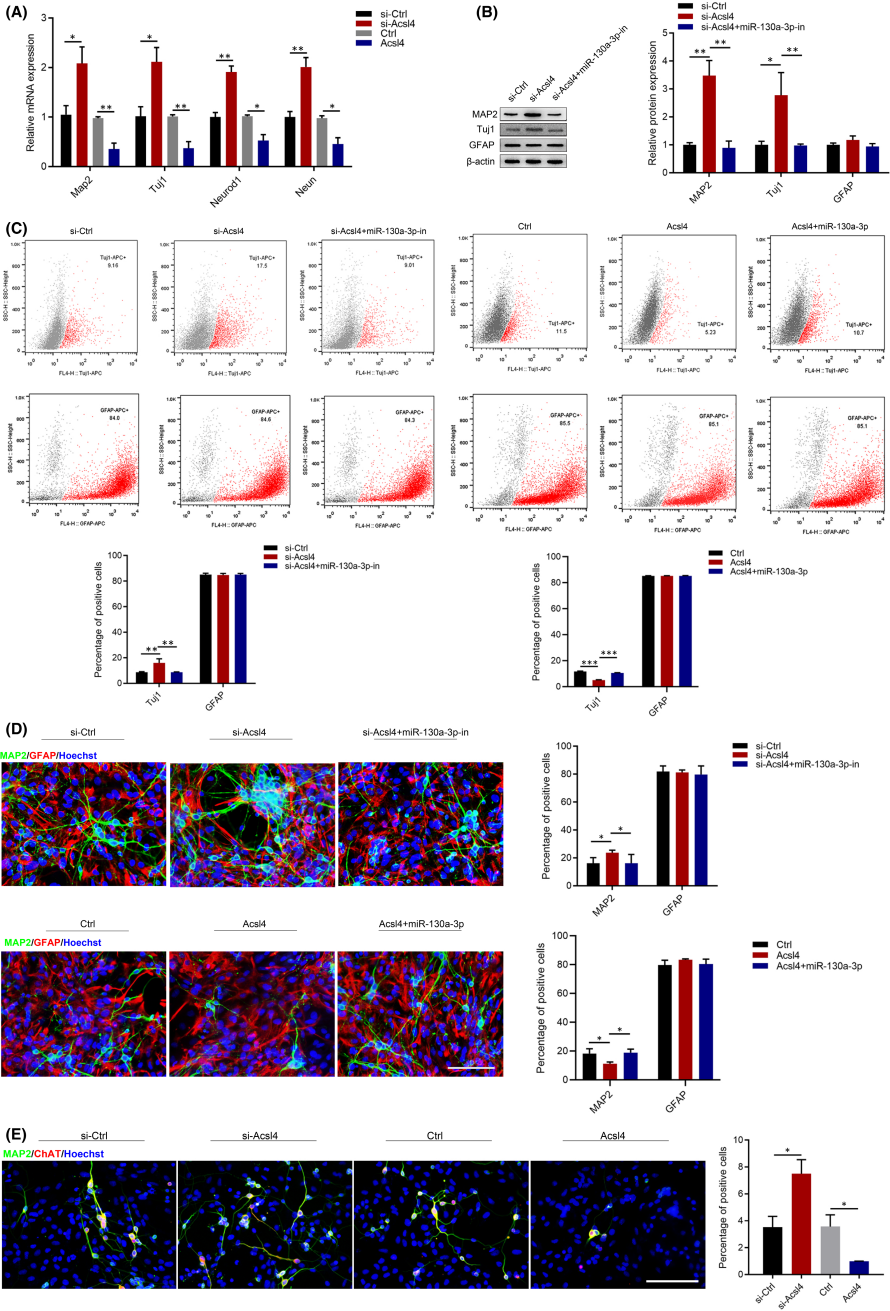
miR‐130a‐3p regulates differentiation of NSCs by targeting *Acsl4*. To study the effect of miR‐130a‐3p‐*Acsl4* on NSCs, they were transfected with *Acsl4* siRNA and overexpression plasmid, or miR‐130a‐3p/NC mimics and miR‐130a‐3p/NC inhibitor for 7d. si‐Ctrl: blank siRNA control group. si‐*Acsl4*: *Acsl4* siRNA group. si‐*Acsl4*+miR‐130a‐3p‐in: *Acsl4* siRNA +miR‐130a‐3p inhibitor–treated group. Ctrl: blank plasmid control group. *Acsl4*: *Acsl4* overexpression plasmid‐treated group. *Acsl4*+miR‐130a‐3p: *Acsl4* overexpression plasmid + miR‐130a‐3p mimic‐treated group. (A) The expression of the neuronal markers, *Map2*, *Tuj1*, *Neurod1* and *Neun*, in NSCs were detected by RT‐qPCR, and using Gapdh as endogenous control. (B) Western blot was performed to detect the expression of the differentiation markers, MAP2, Tuj1 and GFAP, in NSCs. (C) The percentage of Tuj1 and GFAP positive cells detected by flow cytometry assay. (D) Immunofluorescence analysis of MAP2(green) and GFAP (red) positive cells. Scale bars: 100 μm. (E) Immunofluorescence analysis of MAP2 (green) and ChAT (red) positive cells. Scale bars: 100 μm. The results are described as the mean ±SEM at least three separate experiments. *p < 0.05, **p < 0.01, ***p < 0.001 vs. indicated group. (one‐way analysis of variance followed by Tukey's post hoc test)

To further confirm the role of *Acsl4* in NSC proliferation and differentiation, we transfected *Acsl4* siRNA or overexpression plasmid. The inhibition and overexpression of Acsl4 were verified by RT‐qPCR and Western blot (Figure S3). As shown in Figure S4, *Acsl4* had no effect on cell proliferation. The RT‐qPCR results showed that *Acsl4* siRNA significantly increased *Map2*, *Tuj1*, *Neurod1* and *Neun* expression, whereas that *Acsl4* overexpression significantly decreased their expression (Figure 4A). Western blot demonstrated that *Acsl4* siRNA specifically increased the expression of MAP2 and Tuj1, whereas silencing miR‐130a‐3p partially suppressed the expression of these markers. Notably, GFAP expression showed no changes (Figure 4B). Flow cytometry and immunofluorescence showed that the percentage of neurons was notably increased in the si‐*Acsl4* group compared with the si‐Ctrl group. In contrast, the percentage of neurons was partially decreased after miR‐130a‐3p downexpression. Similarly, *Acsl4* overexpression remarkably decreased the percentage of neurons, while neurons were partially increased after overexpression of miR‐130a‐3p. Finally, there were no changes in the number of astrocytes (Figure 4C,D). Immunofluorescence staining of MAP2 and ChAT differentiated cells indicated that there were more MAP2 and ChAT double positive cells in si‐*Acsl4* group, whereas that *Acsl4* overexpression significantly decreased their expression (Figure 4E). Our results revealed that miR‐130a‐3p regulated differentiation of NSCs by targeting *Acsl4*.

### 3.5 miR‐130a‐3p affected the Akt/PI3K Signalling pathway

**FIGURE 5:**
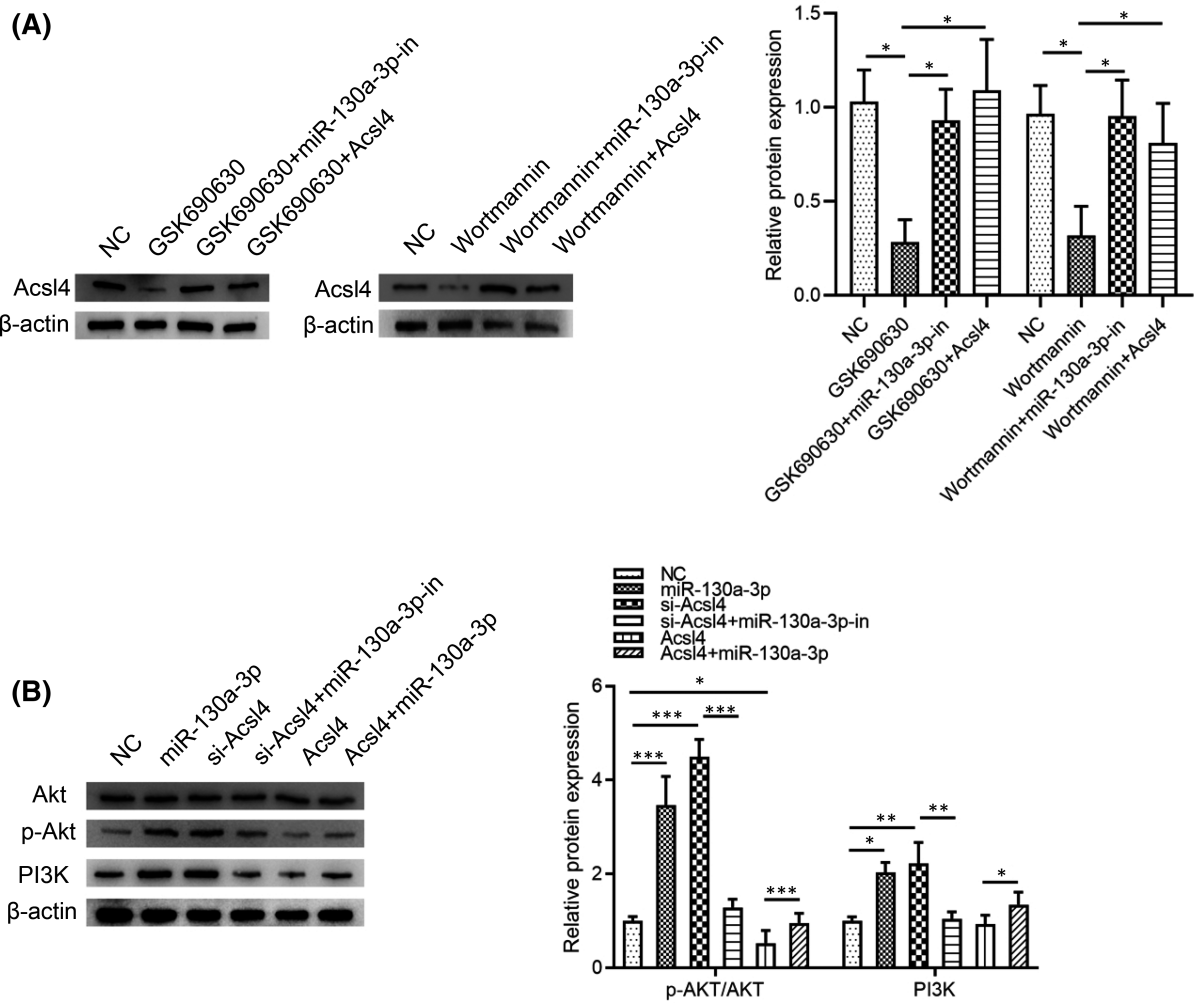
miR‐130a‐3p leads to enhanced Akt/PI3K Signalling pathway. To study the effect of miR‐130a‐3p‐*Acsl4* on Akt/PI3K Signalling pathway in NSCs, they were transfected with *Acsl4* siRNA and overexpression plasmid, or miR‐130a‐3p/NC mimics and miR‐130a‐3p/NC inhibitor for 7d. NC: blank control group. GSK690693: inhibitors of AKT kinases. GSK690693+miR‐130a‐3p‐in: inhibitors of AKT kinases+miR‐130a‐3p inhibitor–treated group. GSK690693+*Acsl4*: inhibitors of AKT kinases+Acsl4 overexpression plasmid‐treated group. Wortmanin: inhibitors of PI3K kinases. Wortmanin+miR‐130a‐3p‐in: inhibitors of PI3K kinases+miR‐130a‐3p inhibitor–treated group. Wortmanin+Acsl4: inhibitors of PI3K kinases+Acsl4 overexpression plasmid‐treated group. (A) Western blot was performed to detect p‐Akt and PI3K. NC: blank control group. miR‐130a‐3p: miR‐130a‐3p mimic–treated group. si‐*Acsl4*: *Acsl4* siRNA group. si‐*Acsl4*+miR‐130a‐3p‐in: *Acsl4* siRNA +miR‐130a‐3p inhibitor–treated group. *Acsl4*: *Acsl4* overexpression plasmid‐treated group. *Acsl4*+miR‐130a‐3p: *Acsl4* overexpression plasmid+miR‐130a‐3p mimic‐treated group. After total protein extraction, Akt, p‐Akt, and PI3K level expression were analysed using Western blot with β‐actin as endogenous control. (B) Western blot was performed to detect p‐Akt/Akt and PI3K expression. The results are described as the mean ± SEM at least three separate experiments. *p < 0.05, **p < 0.01, ***p < 0.001 vs. indicated group. (one‐way analysis of variance followed by Tukey's post hoc test)

Based on previous report that PI3K play an important role in cell growth, survival, differentiation, and metabolism.^21^ Akt, as the main molecule downstream of PI3K,

is a key molecule in growth factor signalling pathways, which regulates neural development.^22^ After the addition of pharmacological inhibitors of kinases (GSK690693, AKT; Wortmanin, PI3K), we found that the level of *Acsl4* were significantly downregulated, but were increased in NSCs silencing miR‐130a‐3p or overexpressing *Acsl4* (Figure 5A). To investigate the regulatory activity of miR‐130a‐3p on the Akt/PI3K signalling pathway, Akt, p‐Akt, and PI3K levels were determined by Western blot (Figure 5B). These results confirmed that the p‐Akt: Akt ratio was specifically increased in cells overexpressing miR‐130a‐3p or cells in which *Acsl4* had been silenced. In contrast, Akt ratio was specifically decreased in cells overexpressing *Acsl4*. Moreover, silencing miR‐130a‐3p partially suppressed the increased p‐Akt, or overexpressing miR‐130a‐3p partially restored the p‐Akt. Consistent with these results, the PI3K levels were increased in cells overexpressing miR‐130a‐3p or in which *Acsl4* had been silenced. The PI3K levels were decreased in cells overexpressing *Acsl4*; additionally, miR‐130a‐3p partially suppressed these changes. These results suggested that miR‐130a‐3p regulated Akt/PI3K signalling pathway.

## 4 DISCUSSION

NSCs are self‐renewing, multipotent progenitors that can differentiate into neurons, astrocytes and oligodendrocytes.^23^ A fundamental understanding of NSC fate determination during neural development will help us to effectively utilize stem cell‐based therapies for neurological diseases. Numerous studies have shown that a kind of small non‐coding RNAs, called miRNAs can precisely regulate the post‐transcriptional expression of target genes and play an important role in NSC activation, proliferation, differentiation and apoptosis.^24^, ^25^ By exploring interactions between miRNAs and the gene regulatory networks that control neurogenesis, we can better understand miRNA‐based regulation, which can eventually be exploited to facilitate treatments for NDs. In our study, we added new evidence for the crucial role of miR‐130a‐3p during neurogenesis. Our results indicated that upregulated miR‐130a‐3p expression promoted NSCs neuronal differentiation of NSCs, but had no effect on proliferation. Thus, we speculate that miR‐130a‐3p may play an important role in NSCs differentiation.

GO analysis of the 67 predicted target genes of miR‐130a‐3p revealed that *Acsl4* was enriched during neuronal differentiation. *Acsl4* is a member of the acyl‐CoA synthetases (ACS) family that has been identified as the main isozyme required for polyunsaturated fatty acid metabolism.^26^, ^27^ Previous studies have reported that *Acsl4* may play roles in intracellular lipid storage,^28^ cholesterol transport,^29^ arachidonic acid metabolism.^30^ Recent studies have indicated that *Acsl4* is downregulated in glioma, where it has anti‐proliferative effects.^31^ Yu et al.^27^ found that forced *Acsl4* overexpression promoted neuronal death, whereas knockdown of *Acsl4* had neuroprotective and anti‐inflammatory effects. Jia et al.^32^ found that *Acsl4* mutants reduced neuroblast proliferation in *Drosophila*. Furthermore, RNA‐seq analysis revealed that cell cycle‐related genes were downregulated and neuronal differentiation genes were upregulated. In this study, we demonstrated that knockdown *Acsl4* promoted the neuronal differentiation of NSCs, whereas silencing miR‐130a‐3p partially suppressed these effects.

miR‐130a‐3p promoted the neuronal differentiation of NSCs, and that this process was dependent on AKT/PI3K signalling pathway. Accumulating evidence has shown that the AKT/PI3K signalling pathway controls NSC self‐renewal and differentiation.^33^ Moreover, it has been confirmed that miR‐130a exerted neuroprotective effects through PI3K/AKT pathway.^19^ However, there were still some limitations to this work. First, the upstream regulatory mechanism that controls miR‐130a‐3p requires further investigation. Second, each miRNA can recognize and inhibit hundreds of mRNA targets, and increasing or decreasing of their expression may eventually lead to a comprehensive change. Therefore, it is unclear whether other target genes are also involved in the regulatory mechanism of miR‐103‐3p in NSCs.

In conclusion, we provided evidence that miR‐130a‐3p regulated the differentiation of NSCs without altering cell proliferation. Mechanistically, miR‐130a‐3p promoted the differentiation of NSCs into neurons by targeting *Acsl4*, and activating PI3K/AKT signalling pathway.

## CONFLICTS OF INTEREST

None.

## AUTHOR CONTRIBUTION

**Wen Li:**Conceptualization (equal); Data curation (equal); Formal analysis (equal); Funding acquisition (equal); Investigation (equal); Methodology (equal); Project administration (equal); Software (lead); Supervision (lead); Validation (lead); Visualization (lead); Writing – original draft (lead); Writing – review & editing (equal). **Bo‐Quan Shan:** Conceptualization (equal); Data curation (equal); Formal analysis (equal); Funding acquisition (equal); Investigation (equal); Methodology (equal); Project administration (equal); Resources (supporting); Software (supporting); Supervision (supporting); Validation (supporting); Visualization (supporting); Writing – original draft (supporting); Writing – review & editing (supporting). **He‐Yan Zhao:** Conceptualization (equal); Data curation (supporting); Formal analysis (supporting); Funding acquisition (supporting); Investigation (supporting); Methodology (supporting); Project administration (supporting); Resources (supporting); Software (supporting); Supervision (supporting); Validation (supporting); Visualization (supporting); Writing – original draft (supporting); Writing – review & editing (supporting). **Hui He:** Conceptualization (supporting); Data curation (supporting); Formal analysis (supporting); Funding acquisition (supporting); Investigation (supporting); Methodology (supporting); Project administration (supporting); Resources (supporting); Software (supporting); Supervision (supporting); Validation (supporting); Visualization (supporting); Writing – original draft (supporting); Writing – review & editing (supporting). **Mei‐Ling Tian:** Conceptualization (supporting); Data curation (supporting); Formal analysis (supporting); Funding acquisition (supporting); Investigation (supporting); Methodology (supporting); Project administration (supporting); Resources (supporting); Software (supporting); Supervision (supporting); Validation (supporting); Visualization (supporting); Writing – original draft (supporting); Writing – review & editing (supporting). **Xiang Cheng:** Conceptualization (supporting); Data curation (supporting); Formal analysis (supporting); Funding acquisition (supporting); Investigation (supporting); Methodology (supporting); Project administration (supporting); Resources (supporting); Software (supporting); Supervision (supporting); Validation (supporting); Visualization (supporting); Writing – original draft (supporting); Writing – review & editing (supporting). **Jian‐Bing Qin:** Conceptualization (supporting); Data curation (supporting); Formal analysis (supporting); Funding acquisition (supporting); Investigation (equal); Methodology (equal); Project administration (supporting); Resources (supporting); Software (supporting); Supervision (supporting); Validation (supporting); Visualization (supporting); Writing – original draft (supporting); Writing – review & editing (equal). **Guo‐Hua Jin:** Conceptualization (equal); Data curation (equal); Formal analysis (equal); Funding acquisition (equal); Investigation (equal); Methodology (equal); Project administration (equal); Resources (supporting); Software (supporting); Supervision (supporting); Validation (supporting); Visualization (supporting); Writing – original draft (supporting); Writing – review & editing (equal).

## Supporting information

Fig S1

Fig S2

Fig S3

Fig S4

Table S1

## References

[1] Slanzi A , Iannoto G , Rossi B , Zenaro E , Constantin G . In vitro models of neurodegenerative diseases. Front Cell Dev Biol. 2020;8:328. 32528949 10.3389/fcell.2020.00328 PMC7247860

[2] Muddapu VR , Dharshini SAP , Chakravarthy VS , Gromiha MM . Neurodegenerative diseases – Is metabolic deficiency the root cause? Front Neurosci. 2020;14:213. 32296300 10.3389/fnins.2020.00213 PMC7137637

[3] Ottoboni L , von Wunster B , Martino G . Therapeutic plasticity of neural stem cells. Front Neurol. 2020;11:148. 32265815 10.3389/fneur.2020.00148 PMC7100551

[4] Andreotti JP , Silva WN , Costa AC , et al. Neural stem cell niche heterogeneity. Semin Cell Dev Biol. 2019;95:42‐53. 30639325 10.1016/j.semcdb.2019.01.005 PMC6710163

[5] Pidikova P , Reis R , Herichova I . miRNA clusters with down‐regulated expression in human colorectal cancer and their regulation. Int J Mol Sci. 2020;21(13). 10.3390/ijms21134633 PMC7369991 32610706

[6] Bofill‐De Ros X , Yang A , Gu S . IsomiRs: Expanding the miRNA repression toolbox beyond the seed. Biochim Biophys Acta Gene Regul Mech. 2020;1863(4):194373. 10.1016/j.bbagrm.2019.03.005 PMC6776719 30953728

[7] Kou X , Chen D , Chen N . The regulation of microRNAs in Alzheimer's Disease. Front Neurol. 2020;11:288. 32362867 10.3389/fneur.2020.00288 PMC7180504

[8] Kumar S , Curran JE , DeLeon E , et al. Role of miRNA‐mRNA interaction in neural stem cell differentiation of induced pluripotent stem cells. Int J Mol Sci. 2020;21(19):6980. 10.3390/ijms21196980 PMC7582477 32977388

[9] Yoo AS , Sun AX , Li L , et al. MicroRNA‐mediated conversion of human fibroblasts to neurons. Nature. 2011;476(7359):228‐231. 21753754 10.1038/nature10323 PMC3348862

[10] Chen ML , Hong CG , Yue T , et al. Inhibition of miR‐331‐3p and miR‐9‐5p ameliorates Alzheimer's disease by enhancing autophagy. Theranostics. 2021;11(5):2395‐2409. 33500732 10.7150/thno.47408 PMC7797673

[11] Wang ZF , Liao F , Wu H , Dai J . Glioma stem cells‐derived exosomal miR‐26a promotes angiogenesis of microvessel endothelial cells in glioma. J Exp Clin Cancer Res. 2019;38(1):201. 31101062 10.1186/s13046-019-1181-4 PMC6525364

[12] Tian X , Fei Q , Du M , et al. miR‐130a‐3p regulated TGF‐beta1‐induced epithelial‐mesenchymal transition depends on SMAD4 in EC‐1 cells. Cancer Med. 2019;8(3):1197‐1208. 30741461 10.1002/cam4.1981 PMC6434193

[13] Wang Z , Li Z , Fu Y , Han L , Tian Y . MiRNA‐130a‐3p inhibits cell proliferation, migration, and TMZ resistance in glioblastoma by targeting Sp1. Am J Transl Res. 2019;11(12):7272‐7285. 31934277 PMC6943444

[14] Zhong G , Lin Y , Wang X , Wang K , Liu J , Wei W . H19 knockdown suppresses proliferation and induces apoptosis by regulating miR‐130a‐3p/SATB1 in breast cancer cells. Onco Targets Ther. 2020;13:12501‐12513. 33324070 10.2147/OTT.S280142 PMC7733342

[15] Jiang X , Ruan XL , Xue YX , Yang S , Shi M , Wang LN . Metformin reduces the senescence of renal tubular epithelial cells in diabetic nephropathy via the MBNL1/miR‐130a‐3p/STAT3 pathway. Oxid Med Cell Longev. 2020;2020:8708236. 32104542 10.1155/2020/8708236 PMC7035567

[16] Yang S , Guo S , Tong S , Sun X . Exosomal miR‐130a‐3p regulates osteogenic differentiation of human adipose‐derived stem cells through mediating SIRT7/Wnt/beta‐catenin axis. Cell Prolif. 2020;53(10):e12890. 32808361 10.1111/cpr.12890 PMC7574877

[17] Glaesel K , May C , Marcus K , Matschke V , Theiss C , Theis V . miR‐129‐5p and miR‐130a‐3p Regulate VEGFR‐2 expression in sensory and motor neurons during development. Int J Mol Sci. 2020;21(11):3839. 10.3390/ijms21113839 PMC7312753 32481647

[18] He H , Li W , Peng M , et al. MicroRNA expression profiles of neural stem cells following valproate inducement. J Cell Biochem. 2018;119(7):6204‐6215. 29575035 10.1002/jcb.26831

[19] Zheng T , Shi Y , Zhang J , et al. MiR‐130a exerts neuroprotective effects against ischemic stroke through PTEN/PI3K/AKT pathway. Biomed Pharmacother. 2019;117:109117. 10.1016/j.biopha.2019.109117 31226635

[20] Ferreira‐Vieira TH , Guimaraes IM , Silva FR , Ribeiro FM . Alzheimer's disease: targeting the cholinergic system. Curr Neuropharmacol. 2016;14(1):101‐115. 26813123 10.2174/1570159X13666150716165726 PMC4787279

[21] Xu F , Na L , Li Y , Chen L . Roles of the PI3K/AKT/mTOR signalling pathways in neurodegenerative diseases and tumours. Cell Biosci. 2020;10:54. 32266056 10.1186/s13578-020-00416-0 PMC7110906

[22] Hemmings BA , Restuccia DF . PI3K‐PKB/Akt pathway. Cold Spring Harb Perspect Biol. 2012;4(9):a011189. 10.1101/cshperspect.a011189 PMC3428770 22952397

[23] Liu S , Chen Z . Employing endogenous NSCs to promote recovery of spinal cord injury. Stem Cells Int. 2019;2019:1958631. 31191666 10.1155/2019/1958631 PMC6525819

[24] Zammit V , Baron B , Ayers D . MiRNA influences in neuroblast modulation: an introspective analysis. Genes (Basel). 2018;9(1):26. 10.3390/genes9010026 PMC5793179 29315268

[25] Stappert L , Klaus F , Brustle O . MicroRNAs engage in complex circuits regulating adult neurogenesis. Front Neurosci. 2018;12:707. 30455620 10.3389/fnins.2018.00707 PMC6230569

[26] Chen J , Ding C , Chen Y , et al. ACSL4 reprograms fatty acid metabolism in hepatocellular carcinoma via c‐Myc/SREBP1 pathway. Cancer Lett. 2021;502:154‐165. 33340617 10.1016/j.canlet.2020.12.019

[27] Cui Y , Zhang Y , Zhao X , et al. ACSL4 exacerbates ischemic stroke by promoting ferroptosis‐induced brain injury and neuroinflammation. Brain Behav Immun. 2021;93:312. 33444733 10.1016/j.bbi.2021.01.003

[28] Xu X , Gopalacharyulu P , Seppanen‐Laakso T , et al. Insulin signaling regulates fatty acid catabolism at the level of CoA activation. PLoS Genet. 2012;8(1):e1002478. 22275878 10.1371/journal.pgen.1002478 PMC3261918

[29] Duarte A , Poderoso C , Cooke M , et al. Mitochondrial fusion is essential for steroid biosynthesis. PLoS One. 2012;7(9):e45829. 23029265 10.1371/journal.pone.0045829 PMC3448708

[30] Kang MJ , Fujino T , Sasano H , et al. A novel arachidonate‐preferring acyl‐CoA synthetase is present in steroidogenic cells of the rat adrenal, ovary, and testis. Proc Natl Acad Sci U S A. 1997;94(7):2880‐2884. 9096315 10.1073/pnas.94.7.2880 PMC20291

[31] Cheng J , Fan YQ , Liu BH , Zhou H , Wang JM , Chen QX . ACSL4 suppresses glioma cells proliferation via activating ferroptosis. Oncol Rep. 2020;43(1):147‐158. 31789401 10.3892/or.2019.7419 PMC6912066

[32] Jia M , Meng D , Chen M , Li T , Zhang YQ , Yao A . Drosophila homolog of the intellectual disability‐related long‐chain acyl‐CoA synthetase 4 is required for neuroblast proliferation. J Genet Genomics. 2019;46(1):5‐17. 30594466 10.1016/j.jgg.2018.10.006

[33] Lee JE , Lim MS , Park JH , Park CH , Koh HC . S6K promotes dopaminergic neuronal differentiation through PI3K/Akt/mTOR‐dependent signaling pathways in human neural stem cells. Mol Neurobiol. 2016;53(6):3771‐3782. 26143260 10.1007/s12035-015-9325-9

