# Supplementary figures and images for: MiR‐130a‐3p regulates neural stem cell differentiation in vitro by targeting Acsl4
Source: J Cell Mol Med. 2022 Apr 16;26(9):2717–27. doi: 10.1111/jcmm.17285 (PMC9077303; doi:10.1111/jcmm.17285)

*Map2*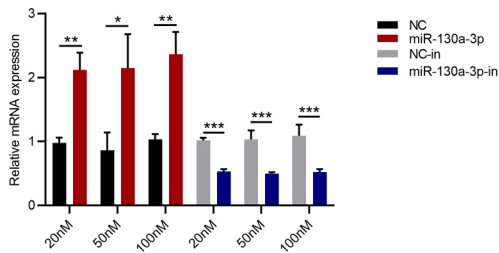*Tuj1*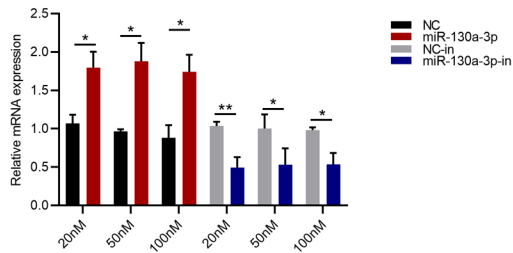*Neurod1*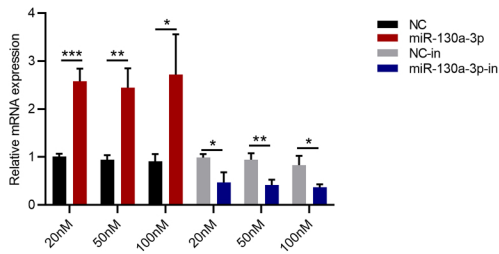*Neun*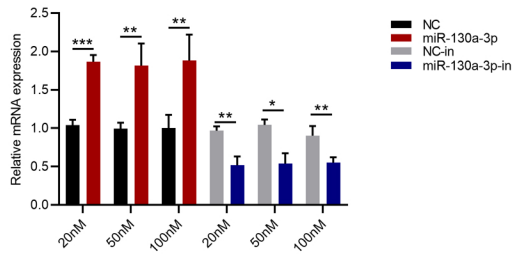*Map2*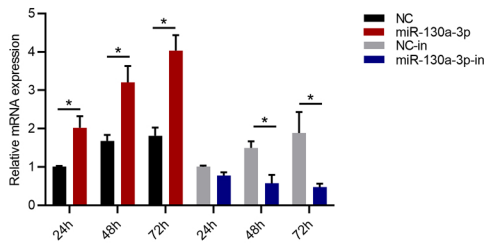*Tuj1*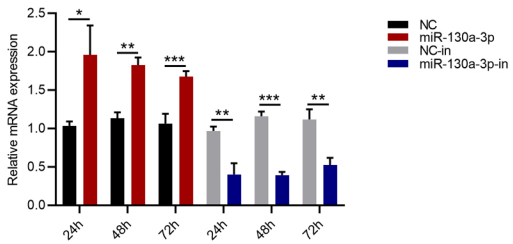*Neurod1*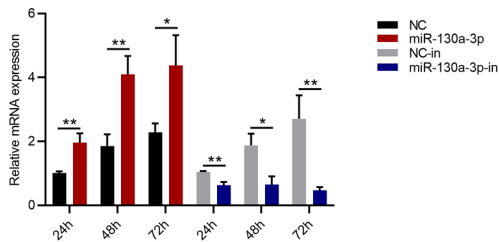*Neun*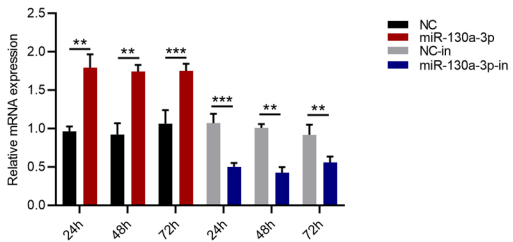

Supplement: Supplementary file 1 — Fig S1 [file JCMM-26-2717-s004.pdf]

## APC-conjugated IgG2A Control

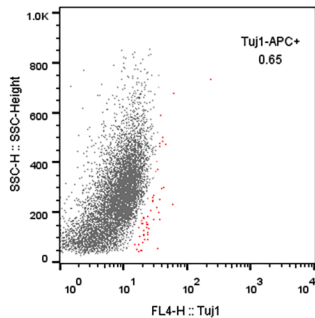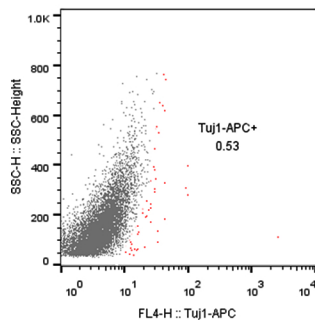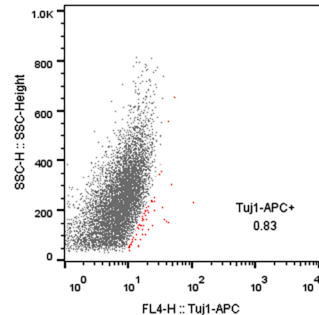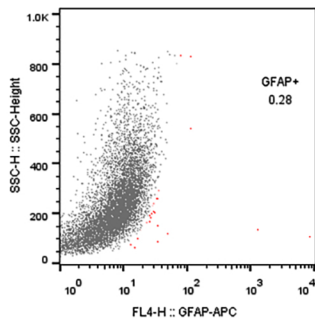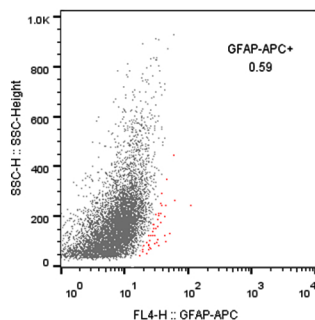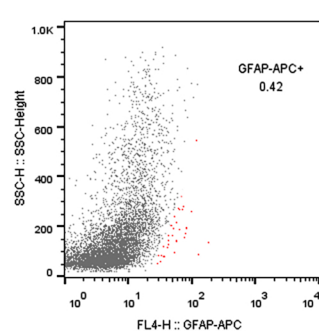

Supplement: Supplementary file 2 — Fig S2 [file JCMM-26-2717-s005.pdf]

A

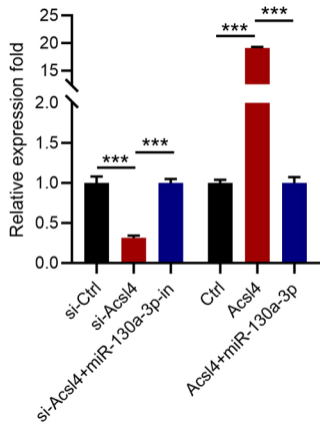

B

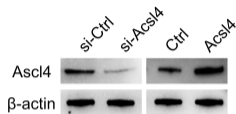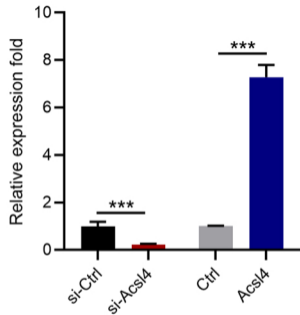

Supplement: Supplementary file 3 — Fig S3 [file JCMM-26-2717-s003.pdf]

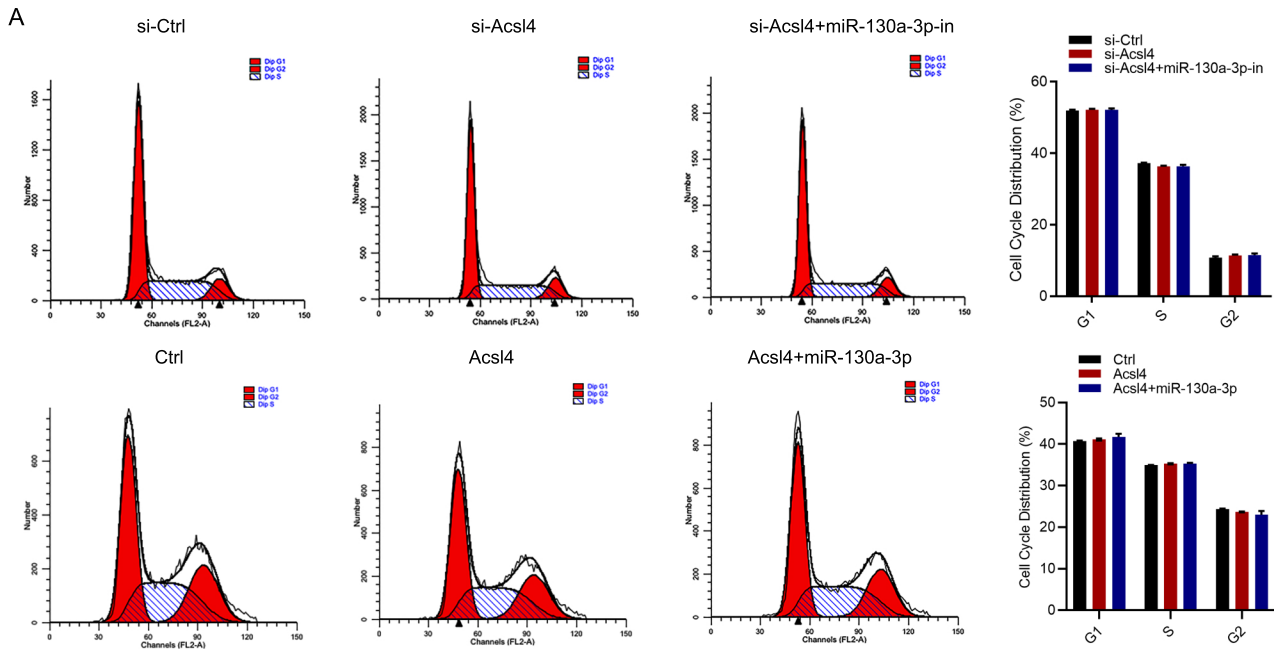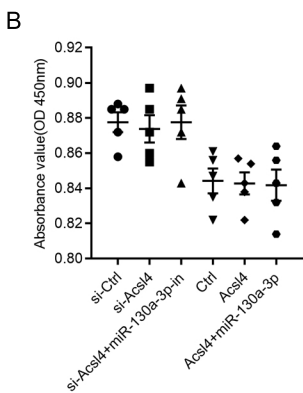

Supplement: Supplementary file 4 — Fig S4 [file JCMM-26-2717-s001.pdf]
